# *Escherichia coli* resistant to the highest priority critically important fluoroquinolone or 3rd and 4th generation cephalosporin antibiotics persist in pigsties

**DOI:** 10.1128/aem.01386-24

**Published:** 2025-05-08

**Authors:** Nicola M. Pfeifer, Michael Weber, Elisabeth Wiegand, Stefanie A. Barth, Christian Berens, Christian Menge

**Affiliations:** 1 Friedrich-Loeffler-Institut, Institute of Molecular Pathogenesis 240210 , Jena, Germany; Universidad de los Andes, Bogotá, Colombia

**Keywords:** *Escherichia coli*, antimicrobial resistance, fluoroquinolones, cephalosporins, fecal organisms, persistence, pig farming

## Abstract

Antimicrobial resistance threatens human and animal health, with antimicrobial usage being a key driver of selection, transmission, and spread of resistant bacteria. Livestock represents a potential reservoir for human transmission, leading authorities to restrict veterinary usage of fluoroquinolones and certain cephalosporins. However, growing evidence indicates that the corresponding resistance determinants can be retained even in the drugs’ absence. To obtain data on the magnitude and dynamics of this phenomenon in pig farming, we quantitatively and qualitatively assessed fluoroquinolone- and cephalosporin-resistant *Escherichia coli* in Thuringian pigsties practicing a closed management system to minimize the impact of externally introduced strains. Pooled fecal samples from consecutive fattening runs at one conventional and two organic farms and from 25 piglet groups from another conventional farm were collected over 16 months and screened for *E. coli* on plates containing enrofloxacin, ceftiofur, or cefquinome. Resistant bacteria were isolated on all farms; their counts varied strongly but were generally higher in piglets and declined with increasing animal age. Phylogenetic comparison of 393 isolates was performed via multiple-locus variable number tandem repeat analysis (MLVA) to follow strain dynamics and persistence. The isolates displayed large phylogenetic heterogeneity, featuring 52 different MLVA patterns. Still, conserved MLVA patterns indicated long-term persistence of specific strains in each farm's environment. This suggests that resistant strains appear well-adapted to the particular farm and its management practices, implying that, beyond restricting usage, further measures, including, e.g., consideration of the type of resistance as well as its persistence and transmission dynamics, will be indispensable to reduce the antimicrobial resistance load in pork production.

**IMPORTANCE:**

Antimicrobial resistance (AMR) represents a global threat to human and animal health, with animals considered a reservoir for transmission of AMR to humans. Because antimicrobial usage is a driver for resistance, one approach to decrease the AMR burden is to reduce its usage. However, this can, but does not necessarily, lead to lower AMR prevalence. German and EU legislation restrict the use of fluoroquinolones and certain cephalosporins, substance classes designated as highest priority critically important antimicrobials for human medicine, in animal husbandry. Longitudinal sampling of organic and conventional farms in Thuringia for resistance to these antibiotic classes revealed that certain resistant *Escherichia coli* strains can persist in the farm environment over extended time periods. These strains displayed farm specificity, indicating adaptation to the particular farm and its management practices, so that their elimination might be difficult, requiring either procedures acting generally against Enterobacterales or targeted action against the specific strains.

## INTRODUCTION

Antimicrobials have contributed considerably to the progress of modern medicine during the last 80 years. However, in the meantime, their usage, including overuse and misuse, has been recognized as a leading driver of antimicrobial resistance (AMR) (1). AMR has accompanied antibiotic use from the beginning (2, 3) and, due to high selective pressure and efficient dissemination, is now considered one of the top 10 global threats to both human and animal health (4), resulting in treatment complications (5, 6), significant economic burden (7, 8), and a substantial number of attributable and associated deaths (9). Strategies to combat AMR include, among others, new antimicrobial drugs, alternative therapies, stewardship programs to preserve drug efficacy, and regulating usage (10, 11). As antimicrobial usage has been linked to AMR emergence and maintenance (12), a major focus is to reduce the amounts of antibiotics consumed (13, 14), especially in the livestock sector (15, 16), which is considered a reservoir for AMR (17–19). However, reducing or banning the use of an antibiotic can (15), but does not necessarily, lead to a concomitant reduction in the prevalence of the corresponding AMR phenotype in agricultural production (20–22), due to different and diverse mechanisms, including cross-resistance, co-selection (23), low to no fitness costs (24, 25), efficient colonization of resistant strains (26, 27), resistance gene presence on promiscuous mobile genetic elements (28, 29), or transmission and circulation within the farm environment and the animal groups present (30, 31). The ubiquitous species *Escherichia coli*, a common, mainly commensal, colonizer of the animal gut, is an excellent model organism to study such AMR dynamics and persistence. It displays high genome plasticity (32, 33), represents a reservoir of AMR determinants (34), and serves as an indicator organism in many official surveillance systems for AMR (35), the latter providing an extensive data basis of the international resistance situation. Important antibiotic classes monitored herein include fluoroquinolones and 3rd and 4th generation cephalosporins. They have been classified as “highest priority critically important antimicrobials” for human medicine (36) and the European Medicines Agency places them in the “restricted” category , which contains substances that should only be used for the treatment of clinical conditions when there are no alternative antibiotics in the lower categories “caution” and “prudence” that could be clinically effective (37). EU (38) and German legislation restricted veterinary usage of fluoroquinolones and 3rd and 4th generation cephalosporins, reducing their sales figures in Germany, which suggests less usage, concomitantly raising the question of whether this would also reduce the corresponding AMR. As fluoroquinolone resistance is mainly mediated by chromosomal mutations and, therefore, transmitted vertically, whereas resistance to cephalosporins is primarily mediated by plasmid-encoded β-lactamases and, thus, additionally transmitted horizontally, studying resistance dynamics to these drugs is also mechanistically interesting. Pigs were chosen as model livestock species because of all meat consumed in Germany and Europe, the percentage of pork meat consumed (Germany: 65%; Europe: 48%) is larger than that of meat from any other livestock species (FAOSTAT/Data/Production/Crops and livestock products/Livestock primary-meat/Production Quantity/2021; data accessed on 16 November 2023) and because the pig production cycle has been considered to represent a reservoir for AMR (39, 40). We therefore longitudinally sampled organic and conventional pig farms, each deploying a farrow-to-finish production system, in Thuringia to determine prevalence and dynamics of fluoroquinolone- or 3rd and 4th generation cephalosporin-resistant *E. coli* over longer time periods on the farms and investigate if this persistence could be traced back to certain strains.

## RESULTS

### Farm and hygiene management on the selected farms differ significantly

The four farms (organic farms O1 and O2, conventional farms C1 and C2) differed fundamentally in their sizes as well as in the structure of their barn buildings, in the organization of the animal groups, the hygiene management practiced, and the selection of the antibiotic agents used. While C2 was the farm housing the most animals, farms C1 and O2 were similar in the number of animals kept, despite their different operational type. Farm O1 was by far the smallest farm and was also the only farm that kept all pig age groups in one common farm building. The separation of different age groups was pursued somewhat more strictly on the conventional farms than on the organic farms. While barns on the conventional farms were disinfected before housing a new group of animals, this was only done for certain age groups or under certain circumstances on the organic farms. At the same time, on the conventional farms, more different antibiotic agents were used during the sampling period. For further details on farm and hygiene management, see Table S1.

### Bacterial growth was frequent on the selective plates from all farms

All samples obtained from the four farms at all sampling time points for quantitative analysis amounted to 92 pooled fecal samples (Table 1; Fig. S1A through D), which were processed and plated on 276 selective plates (farm O1: *n* = 63, O2: *n* = 78, C1: *n* = 78, C2: *n* = 57). Colonies of γ-proteobacteria were detected on 239 selective plates (87%) with 85% (78/92), 79% (73/92), and 96% (88/92) of the GE (Gassner agar containing 4 µg/mL enrofloxacin, a fluoroquinolone), GQ (Gassner agar containing 8 µg/mL cefquinome, a 4th generation cephalosporin), and GF (Gassner agar containing 4 µg/mL ceftiofur, a 3rd generation cephalosporin) plates showing bacterial growth, respectively. Cephalosporin-resistant or fluoroquinolone-resistant γ-proteobacteria (CSRG/FQRG) were, thus, detected on all farms.

**TABLE 1:** Age of the sampled animals at the sampling time points

Age group	Sampling age	Definition of the time point of sampling	Number of samples taken per farm			
			O1	O2	C1	C2
Suckling piglet	At the end of the weaning period	Shortly before weaning or on the day of weaning	7	10^*a*^	26	7
Weaner	At the end of the rearing period	Shortly before transfer to the fattening pen	4	4	n.t.^*b*^	3
Early fattening	At the beginning of the fattening period	1–2 weeks after being placed in the fattening house	3	4	n.t.	3
Mid-fattening	After about half of the fattening period	Depending on the type of farm, after 6–8 weeks (C2) or after 9–12 weeks in the fattening house (O1, O2)	4^*c*^	4	n.t.	3
Late fattening	At the end of the fattening period	In the last days before the first animals from the fattening group were transported to the slaughterhouse	3	4	n.t.	3
Total number of samples taken per farm			21	26	26	19

^*a*^ Does not include the additional suckling piglet sample (suckling piglets 3 sorted out – animals were added to the animals of fattening run 4), which was only analyzed qualitatively.

^*b*^ n.t., none taken.

^*c*^ One sample was taken from a group of pigs in which animals from an early fattening time point and from a mid-fattening time point had been mixed.

### Percentages of resistant bacteria differ strongly between farms and age groups

When comparing the extent of CSRG/FQRG growth on the plates between the farms, specific differences were observed. Every selective plate with samples from farm C2 showed colony growth (57/57), whereas growth was detected on 97% (76/78) and 94% (59/63) of the selective plates from farms C1 and O1, respectively. On farm O2, only 60% (47/78) of the selective plates harbored bacterial colonies. CSRG/FQRG were detected in all age groups, although fewer selective plates showed bacterial growth with increasing age of the animals. For example, samples from suckling piglets led to growth on 141 of the 150 (94%) selective plates, while samples taken at the late fattening stage yielded growth on only 21 of 30 (70%) selective plates. Similar differences between the age groups were also observed when the relative number of colony-forming units (CFUs) of resistant bacteria was calculated with respect to all CFUs on the GA plate (Gassner agar without antibiotics) representing the percentage of all resistant bacteria (the corresponding CFUs are displayed in Table S2). When considering the distribution of these percentage values for all age groups, the upper quartile always remains below 10% (Fig. S2). During the fattening stage, the upper quartile was even below 1% in all samples. However, the values determined cover a broad range, as values between 0% and above 10% were observed in each age group. From suckling piglets to early fattening pigs, individual pooled fecal samples yielded colony counts for the fluoroquinolone- or cephalosporin-resistant population, which accounted for at least 70% of the population on the non-selective GA plate (*n* = 11 samples). Notwithstanding, the median value changed only moderately across the different age groups and was always between 0.1% and 1%, except for the mid-fattening samples, where it was even lower.

**Fig 1:**
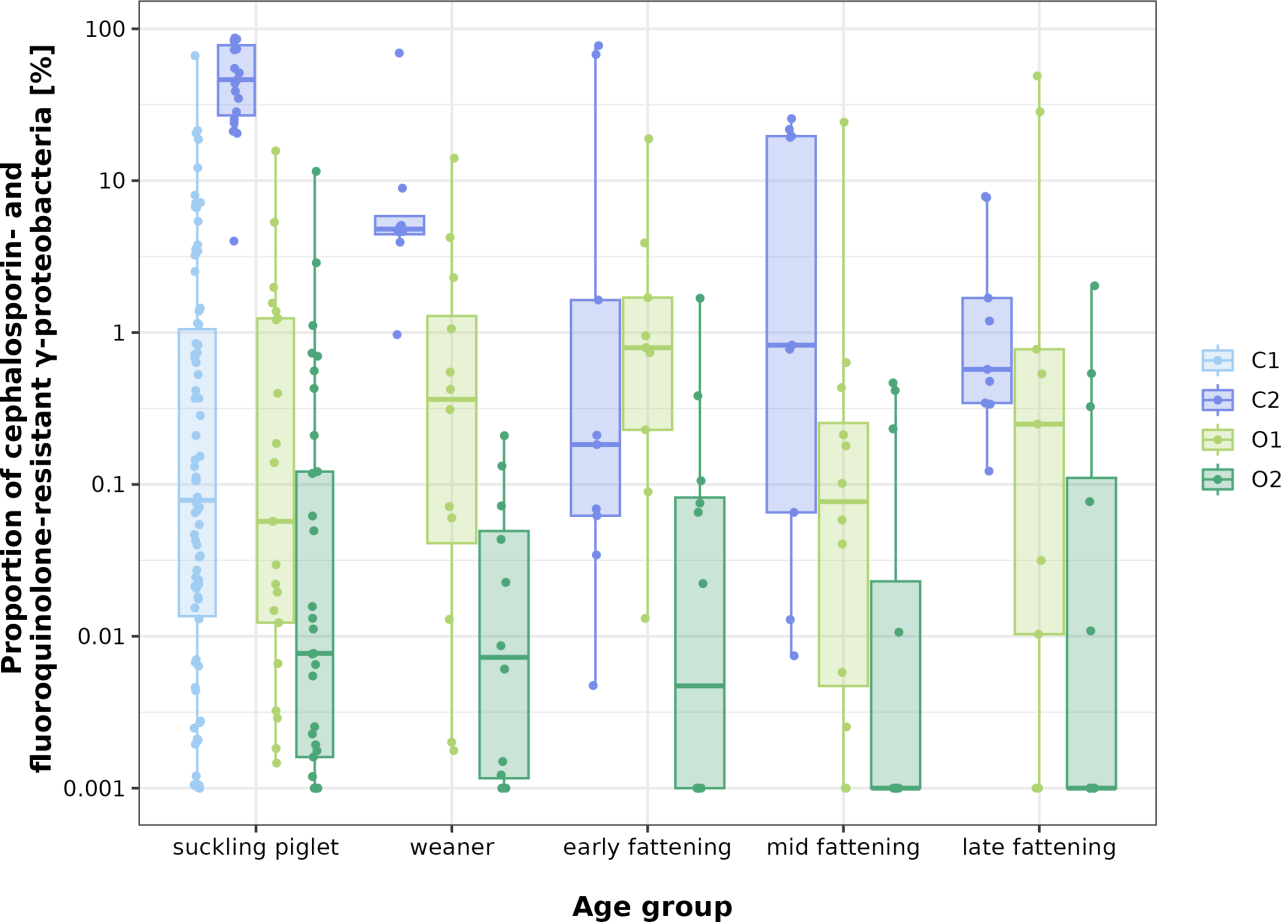
Proportion of cephalosporin-/fluoroquinolone-resistant γ-proteobacteria in all γ-proteobacteria per age group and farm. Representation of all determined percentage values of resistant bacteria on the selective plates as box-and-whisker plots; value points slightly scattered horizontally for better visibility; antennas enclose all values within 1.5 times the interquartile range. In order to be able to logarithmically represent values of pooled fecal samples without growth (measured value 0), the value 0.001 was added to all percentage values of resistant CFU. Values above 100% were truncated at 100% (*n* = 3).

**Fig 2:**
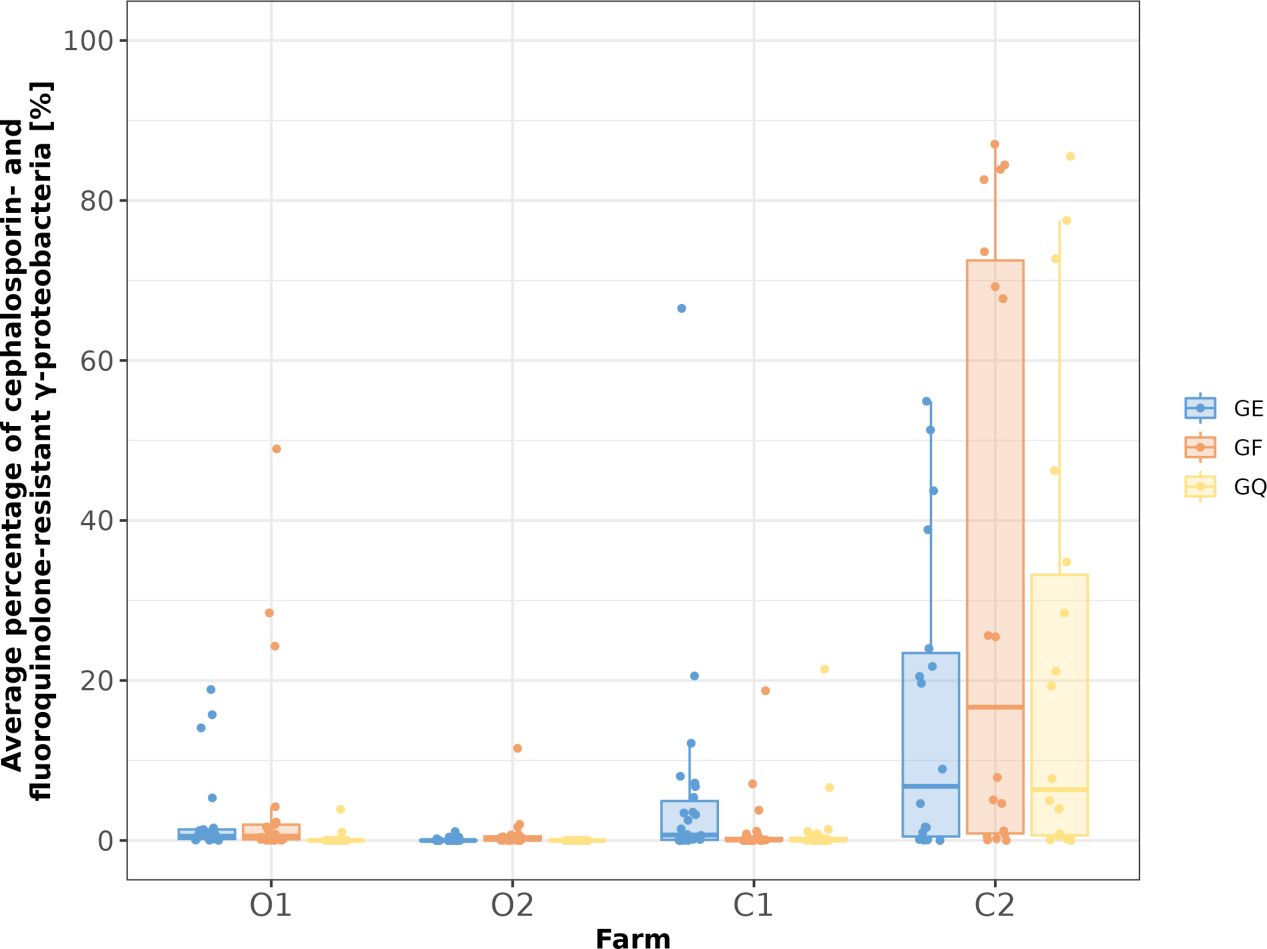
Average percentage of resistant bacteria disaggregated for selective plate and farm. Representation of all percentage values determined for resistant bacteria per selective plate and farm as box-and-whisker plots; value points are slightly scattered horizontally for better visibility; antennas enclose all values within 1.5 times the interquartile range. Values above 100% were excluded (*n* = 3, all on farm C2). GE = Gassner agar supplemented with 4 µg/mL enrofloxacin; GF = Gassner agar supplemented with 4 µg/mL ceftiofur; GQ = Gassner agar supplemented with 8 µg/mL cefquinome. The confidence intervals (95%) for the respective median values are presented in Table S4. Significant differences between the average percentage values of the different farms for the selective plates are not shown in the figure, as they would interfere with its clarity, but are presented in Table S3.

Although CSRG/FQRG were detected in almost all samples, they mainly accounted for less than 10% of the total number of colonies on the corresponding non-selective GA plate (in 86% of the samples). However, the number of resistant colonies also varied within a farm and even between the samples within an age group from one farm (between two [late fattening pigs in C2] and almost five [suckling piglets in C1] powers of 10, Fig. 1). Comparing the four farms with respect to the percentages of enrofloxacin-, ceftiofur-, or cefquinome-resistant bacteria (for CFU counts, see Table S2), significant differences were frequently observed (Fig. 2; Table S3). Only three comparisons did not differ significantly. These were fluoroquinolone-resistant bacteria on farms O1 and C1, C1 and C2, and ceftiofur-resistant bacteria on farms O2 and C1. Weakly significant (0.01 < *P* < 0.05) differences were observed for enrofloxacin-resistant bacteria on farms O1 and C2 as well as for cefquinome-resistant bacteria on farms O1 and C1. All other comparisons yielded highly significant (*P* < 0.001) differences in cell counts of resistant bacteria. Noteworthy is also that the CFU counts on the GF and GQ plates differed significantly for the two organic farms but not for both conventional farms (Table S3). This is also reflected in the distribution of the farm medians of CSRG/FQRG calculated for the samples from suckling piglets (Fig. 1): While the median was below 0.1% at suckling piglet age in the farms C1, O1, and O2, the median of farm C2 was above 50% in the samples from this age group. Strong differences in the values of the farms decreased with increasing age of the animals sampled, leading to median values below 1% at the fattening stage in all farms. Across all age groups, farm O1 showed continuously low numbers of CSRG/FQRG, with only individual samples displaying higher percentage values of resistant bacteria. For C1, only the suckling piglet age values could be compared with the other farms. This range of values resembled that of farm O1, with the higher number of pooled fecal samples from C1 most likely resulting in a wider range of values. Farm O2 consistently had the lowest values of resistant bacteria, since colony growth on this farm’s selective plates only once exceeded 2% of that on the GA plate. The CFU values of farm C2 were frequently among the highest recorded across all farms and age groups, except for the early fattening samples.

### Counts of CSRG/FQRG vary strongly in samples within a farm

**Fig 3:**
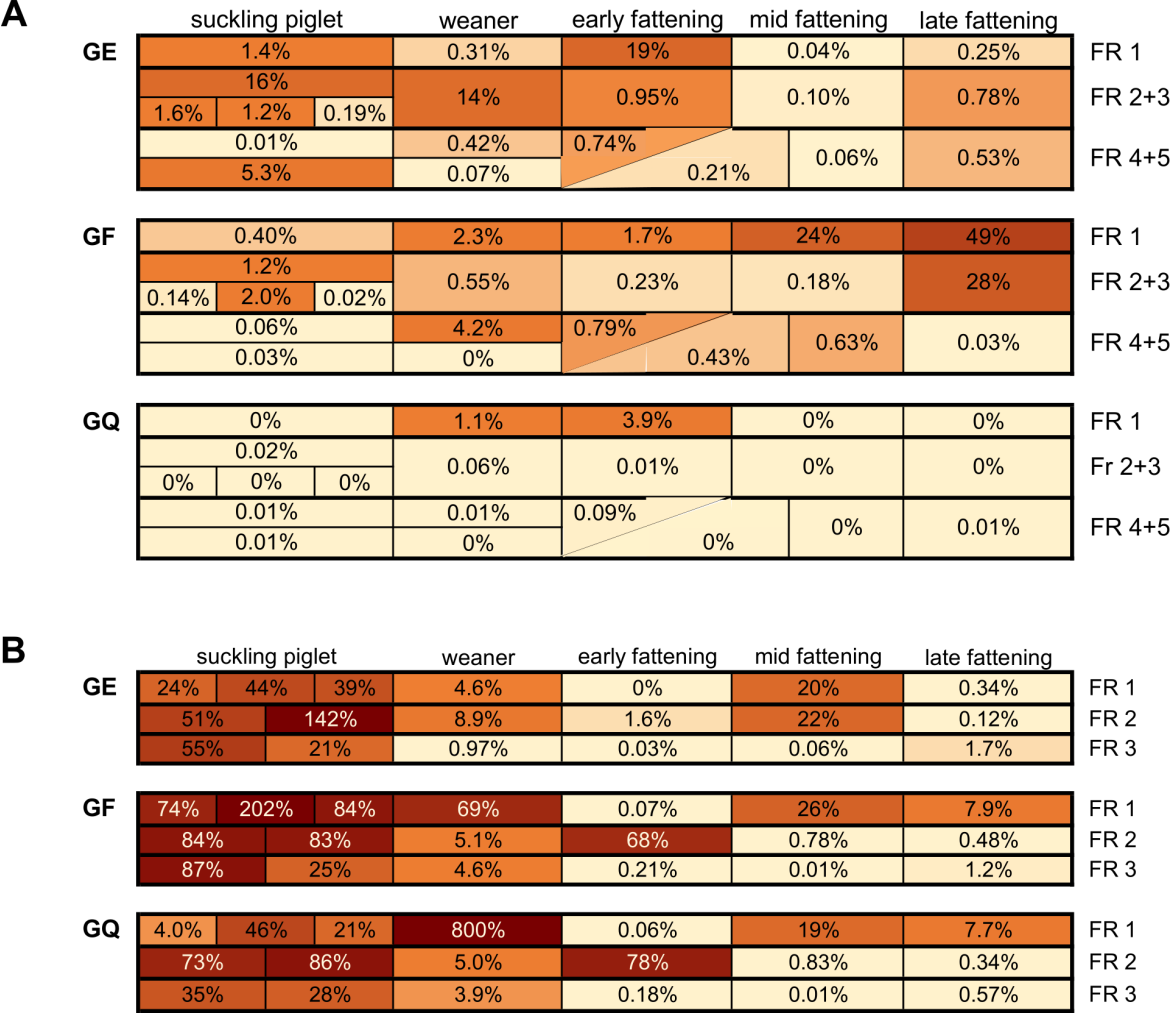
A + B: Percentage values of resistant bacteria on GE, GF, and GQ plates of all fecal samples from farms O1 (**A**) and C2 (**B**) compared between selective plate types. For a detailed description of the structure of the heatmaps, see Fig. S1A-D; GE = Gassner agar supplemented with 4 µg/mL enrofloxacin; GF = Gassner agar supplemented with 4 µg/mL ceftiofur; GQ = Gassner agar supplemented with 8 µg/mL cefquinome; FR = fattening run. The corresponding respective absolute CFU counts are given in Table S2. “0%” corresponds to less than 0.01% of the colonies detected on the Gassner agar plate without antibiotics.

Analyzing the data from individual fattening runs on the four farms, a very heterogeneous picture emerged on farm O1, as the fraction of CSRG/FQRG varied strongly—not just between fattening runs but also within a fattening run (Fig. 3A; absolute numbers are shown in Table S2). The percentages of ceftiofur-resistant bacteria were highest at the late fattening stage of fattening runs 1 and 2 + 3. Higher values were also found in the samples from suckling piglets to the early fattening stage on the GE plates. The distribution of the CSRG did not correspond with that of the FQRG. Only a few colonies were detected on the GQ plates of farm O1.

While almost no cefquinome-resistant bacteria were detected on farm O2, low numbers of ceftiofur-resistant bacteria were consistently observed (Fig. S3A). The fraction of enrofloxacin-resistant bacteria exceeded 0.1% in only a few (4/26) pooled fecal samples.

On farm C1, the CFU counts on the GE plates encompassed at least 1% of the CFU counts on the respective non-selective GA plate in 40% of the samples. The proportion of FQRG almost always outnumbered the proportion of CSRG. Particularly in the first two suckling piglet runs, the fraction of FQRG was well above 1% (8/10; Fig. S3B) in all housing compartments, while in the subsequent suckling piglet runs, such values were detected less frequently (4/16) and only in compartments 3–5. The proportion of ceftiofur- or cefquinome-resistant bacteria was elevated in all compartments only for the first two suckling piglet runs. The percentages of resistant bacteria on the plates supplemented with these two antibiotics were mostly similar.

On farm C2 (Fig. 3B), the percentage values of resistant bacteria were very high in the suckling piglet samples of all fattening runs and in the weaner samples from fattening run 1. In two of these samples, CFU counts were higher on the selective plates than on the GA plate (GE: suckling piglet sample 2b; GF: suckling piglet sample 1b; see Fig. S1D). The GQ plate with the weaner pig sample of fattening run 1 yielded the highest percentage value of all farms, but hardly any CSRG were found in the subsequent sample from the early fattening pigs. The fractions of CSRG/FQRG also varied strongly at the later sampling time points of the fattening runs. However, colony growth on the plates with ceftiofur and cefquinome correlated mostly. This was especially the case for the samples from suckling piglets but also in the samples from the weaners in fattening runs 2 and 3 and, in fattening run 1, in samples from the mid-fattening pigs. In contrast, the differences in resistant bacterial fractions were more pronounced between the fattening runs: Although the highest percentage values were detected in individual samples from fattening run 1, fattening run 2 yielded high values throughout, whereas in fattening run 3, only samples from the suckling piglets scored high. Nevertheless, a decrease in the fraction of resistant bacteria was observed in all fattening runs on this farm until the time of slaughter.

### Despite high diversity in the MLVA profiles, conserved clusters were identified

A total of 393 resistant *E. coli* (CSRE/FQRE) were isolated from 64 pooled fecal samples (Table 2). Up to 16 *E. coli* strains, as defined in the taxonomic sense (41), were isolated from all selective plates per fecal sample. On average, more *E. coli* strains were isolated per fecal sample from a conventional farm than from an organic farm. Two hundred *E. coli* strains were isolated from GE plates, 99 from GF plates, and 94 from GQ plates.

**TABLE 2:** Numbers of individual cephalosporin- and fluoroquinolone-resistant *E. coli* strains isolated per age group and farm and selected for further analysis

Farm	Suckling piglet		Weaner		Early fattening		Mid fattening		Late fattening		Total	
	[*n*]	(%)^*a*^	[*n*]	(%)	[*n*]	(%)	[*n*]	(%)	[*n*]	(%)	[*n*]	(%)
C1	92	(23)	n.t.^*b*^		n.t.		n.t.		n.t.		92	(23)
C2	57	(15)	23	(5.9)	18	(4.6)	18	(4.6)	26	(6.6)	142	(36)
O1	29	(7.4)	23	(5.9)	14	(3.6)	24	(6.1)	21	(5.3)	111	(28)
O2	24	(6.1)	7	(1.8)	1	(0.25)	8	(2.0)	8	(2.0)	48	(12)
Sum	202	(51)	53	(13)	33	(8.4)	50	(13)	55	(14)	393	(100)

^*a*^ Percentage refers to all 393 isolated *E. coli* strains.

^*b*^ n.t., none taken.

The reduced numbers of resistant CFUs at the later sampling time points in the fattening runs could be due to reduced numbers of bacteria belonging to different resistant strains or due to a reduction in the number of resistant strains present in the animals. In an attempt to distinguish between these two possibilities, we assayed strain phylogeny by performing multiple-locus variable number tandem repeat analysis (MLVA) and identified 57 different MLVA profiles (MPs) among the 393 isolates (Fig. S4). In multiple cases, several strains had the same or very similar MPs, so that they were grouped into a MLVA cluster, while the MPs of 18 strains occurred only once in the entire data set. On average, 6.9 strains had the same MP. Farms C2 and O2 had a rather low uniformity index (4.2 and 3.2, respectively), indicating greater strain diversity, whereas the uniformity index of farms O1 and C1 was relatively high with 8.5 and 9.2, respectively.

**Fig 4:**
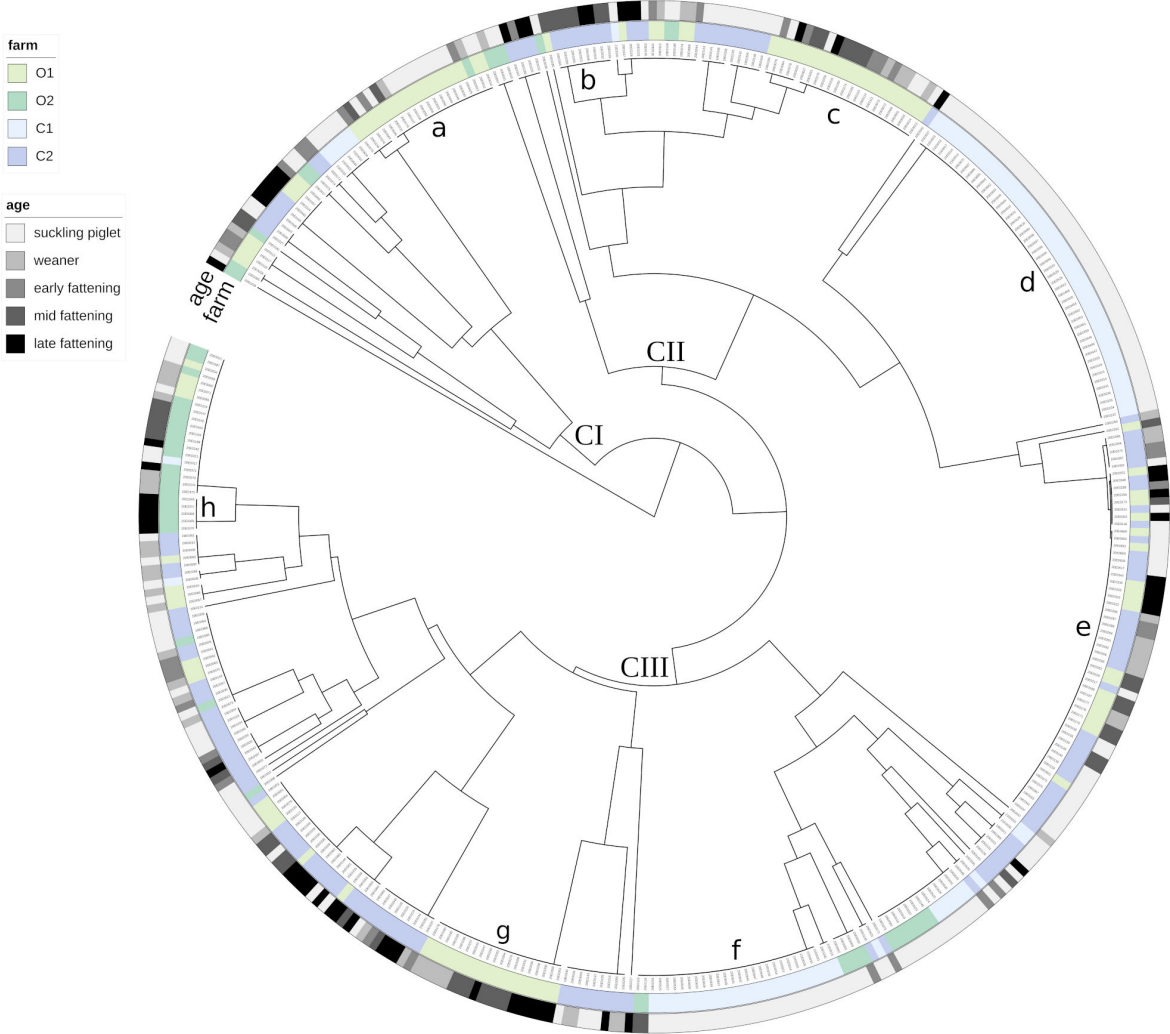
Phylogeny tree calculated from the MLVA profiles determined for the 393 strains isolated. CI–CIII = Three clades, in which exemplary MLVA clusters have been named (letters “a” to “h”). The comparison of MLVA profiles is based on the Dice coefficient. Clusters were created with unweighted pair group method with arithmetic mean.

The MPs detected and the MLVA clusters derived were assigned to three clades (Fig. 4). The largest MLVA cluster was composed of 55 strains from two farms (O1 and C2, MP-e). Many MLVA clusters included strains from only one farm (MP-a, MP-c, and MP-g from farm O1; MP-h from farm O2; MP-d and MP-f from farm C1; MP-b from farm C2), i.e., the MP distribution was primarily farm-specific. In many instances, the associated strains belonged to samples from different age groups (Fig. 4: gray gradation in the outer ring) and fattening runs, or they were isolated from different barn compartments. Nonetheless, despite screening for different phenotypes while picking strains, several *E. coli* strains from one fecal sample or even from the same selective plate did also display the same MP (for instance, six strains from one fecal sample from farm O1).

### MLVA clusters allow the identification of persistent strains

**Fig 5:**
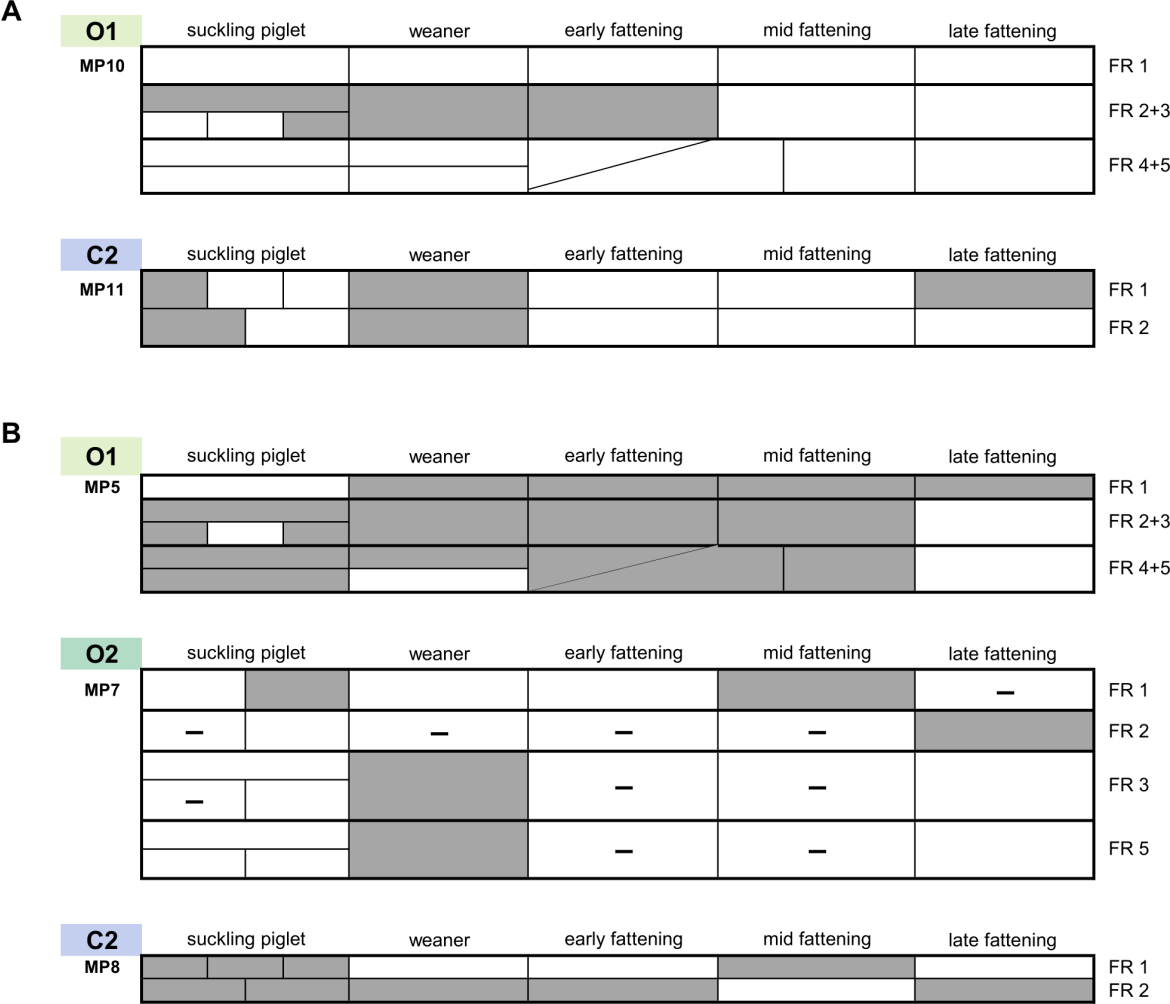
Examples of animal group-persistent (**A**) and farm-persistent (**B**) MLVA profiles. For a detailed structure of the heatmaps, see Fig. S1A-D; MP = MLVA profile (indicated on the left); FR = fattening run; gray color = at least one isolate from this pooled fecal sample with corresponding MP detected; farms marked in color; – = no cephalosporin-resistant/fluoroquinolone-resistant *E. coli* were isolated from this pooled fecal sample.

If strains assigned to the same MLVA cluster were detected in at least three different age groups in the same fattening run, these strains were considered as “persistent” within a fattening run or as “animal group-persistent.” In farm O1, six of the 13 MPs (46%) and in farm C2, seven of the 34 MPs (21%) were persistent within a fattening run (Fig. 5A). In contrast, in farm O2, no strain with a given MP met this criterion. Since no complete fattening runs had been sampled in farm C1, animal group-persistent strains could not be identified.

When strains assigned to the same MLVA cluster were detected in the fecal samples of at least three different age groups per fattening run and additionally at least in one fecal sample of another fattening run, the strains belonging to this MLVA cluster were considered “farm-persistent” (MP5 from O1, MP8 from C2; Fig. 5B). Likewise, strains of a MLVA cluster were designated as persistent on the farm if the corresponding MP was detected at least once in each of three or more fattening runs (MP7 from O2 and MP13 from O1). In farm O1, six of the 13 MPs (46%) were farm-persistent. Five of these six MPs also met the criterion of animal group persistence. On farm O2, three of the 15 MPs (20%) occurred persistently on the farm without being animal group-persistent. From farm C2, five of the 34 MPs (15%) were classified as farm-persistent, all of which were also animal group-persistent. For farm C1, farm persistence was defined differently, i.e. as the presence of a MP in at least one strain from more than one-third of the samples that were analyzed qualitatively. Based on this criterion, two of the ten MPs were classified as farm-persistent (20%). Both were detected in 14 of 16 qualitatively analyzed pooled fecal samples.

The strains defined as persistent on the basis of their MP are by definition chromosomally similar but show some variation in resistance phenotype at the different sampling time points (Fig. 5A: In farm O1, of seven strains displaying MP10, five are CSRE and two are FQRE; in farm C2, of nine strains displaying MP11, seven are resistant to both 3rd or 4th generation cephalosporins and fluoroquinolones, two are CSRE; Fig. 5B: In farm O1, of 23 strains displaying MP5, 22 are FQRE and one is resistant to both 3rd or 4th generation cephalosporins and fluoroquinolones; in farm O2, of six strains displaying MP7, three are CSRE and three are FQRE; in farm C2, of 35 strains displaying MP8, five are FQRE and 30 are resistant to both 3rd or 4th generation cephalosporins and fluoroquinolones. This suggests that there might have been some acquisition or loss of extrachromosomal genetic material in these strains during the sampling period.

In contrast to the animal group- and farm-persistent MPs identified for the 393 *E. coli* strains, we also detected several MPs (34/57; 60%) that occurred only once in all samplings on each respective farm. These were designated as farm-singletons and represented 3/13 MPs (23%) on farm O1, 8/15 MPs (53%) on farm O2, 6/10 MPs (60%) on farm C1, and 17/34 MPs (50%) on farm C2, highlighting the high degree of diversity among the isolated strains.

## DISCUSSION

The present study aimed at getting an insight into the prevalence dynamics of bacteria resistant to fluoroquinolones and/or 3rd or 4th generation cephalosporins in the German pig production system. The CSRE/FQRE were isolated from pooled fecal samples taken on four pig farms with a closed management system (no purchase of weaners from other farms for fattening) and typed by MLVA-PCR to identify clusters of chromosomally related strains. The study design—combining longitudinal sampling, encompassing multiple fattening runs per farm, with selective plating—enabled the detection of persisting resistant strains independent of their pathogenic potential and even when accounting for only a minority population within the intestinal coliform flora (represented in our study by the total non-selective CFU counts). Relating thereto, it is important to consider that the number of resistant bacteria detected was higher because of the selective sampling than it would have been using non-selective sampling (42, 43).

### CSRG and FQRG may persist in pigsties

γ-Proteobacteria with resistance to enrofloxacin, ceftiofur, and/or cefquinome were detected on all farms but with widely varying prevalence. Large variations in ESBL prevalence have been determined for German poultry farms as well as for pig farms (44, 45). Schulz et al*.* were able to correlate different occurrences of FQRE in German livestock farms with the respective fluoroquinolone concentrations in farm dust (46). Repeated sampling of the four farms over a period of up to 13 months, nevertheless, allowed us to visualize the dynamics of resistance prevalence on the individual farm. Although the median prevalence of resistant bacteria was low for all age groups, it still decreased with increasing age of the pigs. This decrease was especially obvious for farm C2 and is consistent with other studies detecting the highest prevalence of resistance to fluoroquinolones (47) or cephalosporins (48, 49) in suckling piglets and the lowest in finisher pigs (50, 51). The decrease in resistance prevalence up to the early fattening stage transitioned into a plateau at low levels for the remaining fattening phase. Similar results were also obtained in a study on FQRE in pigs (47). One reason for the lower resistance prevalence in fattening pigs could be due to a reduced antibiotic treatment frequency in older animals (12). The all-in/all-out housing system practiced primarily on conventional farms may also contribute to the reduced CFU counts of resistant strains (48). In contrast, the greater number of stress factors at rearing age due to castration, weaning, and frequent contact with farm employees probably contributes to increased shedding of resistant bacteria (52). The decrease in resistance prevalence is relevant with respect to the antibiotic resistance surveillance programs established in Germany and Europe, which often focus on sampling at fattening age or even at the slaughterhouse (42, 53, 54). For reasons of practicality, this is the most cost-effective sampling time point and can represent the potential hazard posed by porcine food products in the most direct way (55). However, the present study shows that sampling of fattening pigs does not provide information on the resistance situation at rearing age. Yet, farm-derived resistance determinants or the bacteria carrying them can contribute to antibiotic resistance in human medicine even before the animals are slaughtered, if the former are transferred via manure serving as fertilizer to cropland used for food production (56). In addition, resistance determinants or the bacteria carrying them can be disseminated via insects, birds, and rodents, and resistant bacteria can become established in the environment surrounding a farm (57–61). Also, the antibiotic agents themselves are often still present in active form in manure, as 30%–90% of the antibiotics are not or only partially metabolized in the animal treated and are not completely inactivated by manure treatment. Thus, these antibiotics can still exert selective pressure in the environment and thereby pose a public health problem (62). At first, a high prevalence of resistance at rearing age poses a challenge for veterinary medicine and should additionally be avoided for the sake of the One Health approach (63, 64).

The prevalence dynamics of the mostly plasmid-encoded cephalosporin resistance and the mainly chromosomally encoded fluoroquinolone resistance did not display any clear distinctions. However, it was striking and, so far unexplained, that the quotient values of the GF and GQ plates differed significantly in the organic farms but not in the conventional farms (Table S3). Unveiling the mechanistic basis of this difference will require further investigation. In addition, on the conventional farms, there was a tendency for growth on the GF and GQ plates to correlate with that on the GE plates, which might be explained by the presence of isolates with resistance to both antibiotic classes (cephalosporins and fluoroquinolones). Administration of 3rd/4th generation cephalosporins and fluoroquinolones could contribute to the persistence of resistant strains. However, farms O2 and C1 did not utilize such antibiotics during the entire sampling period (Table S1). On farms O1 (fluoroquinolones and cephalosporins) and C2 (only cephalosporins), they were given more regularly in the beginning of the sampling period, but thereafter, only sporadically (Fig. S5), so that drug usage can have contributed to the maintenance of resistant *E. coli* but cannot have been solely responsible for overall persistence on the farms.

### MLVA profiles indicate persistence of fluoroquinolone-/cephalosporin-resistant *E. coli* strains

Despite selecting for resistance to cephalosporins or fluoroquinolones, the *E. coli* strains showed great phylogenetic diversity, reflected by the large number of different MPs. Accordingly, to reduce the prevalence of cephalosporin- and fluoroquinolone resistance on the sampled farms, a large number of strains with different properties, given the high genetic plasticity of the *E. coli* genome (65, 66), would have to be eliminated from the farm environment. Nevertheless, strains with the same MP were isolated from samples of more than one age group within one fattening run on a farm and on multiple occasions even from pooled fecal samples of further fattening runs on the same farm. This indicates persistence of cephalosporin- and fluoroquinolone-resistant bacterial strains and agrees with data from other studies (23, 30, 67, 68). Animal group- and farm-persistent strain presence was maintained in animal groups despite transfer to other pens or compartments and on farms O2 and C2 even after re-housing to other barns. Therefore, the corresponding strains must have persisted in or on the sampled animals. The fact that ten of 13 animal group-persistent MPs (77%) were simultaneously farm-persistent suggests that certain *E. coli* strains cannot only persist in a group of animals but may also spread effectively throughout the farm. Such prolonged shedding of a strain provides more opportunities for contamination and transmission. However, they could also be highly adaptable strains that spread rapidly and efficiently in the farm environment, which is why *E. coli* has been referred to as a generalist species (64). Because the MPs associated with persistence display a certain farm specificity, we assume that the resistant *E. coli* strains present on each farm are well-adapted to the farm and its respective regimes for feeding and/or hygiene management and antibiotic use. This may be a consequence of sampling on farms with farrow-to-finish housing systems, where new bacterial strains are less likely to become introduced to the farm environment through the purchase of animals. Furthermore, recent studies indicate that the fitness costs of carrying AMR conferring genetic elements may be lower than previously considered (69, 70). Under the conditions applied in modern animal husbandry, no matter whether organic or conventional, such persistent strains will likely not be replaced by susceptible strains following prudent use of antimicrobials.

This is even more of concern, as our study may still underestimate the number of persistent strains and the frequency of occurrence of these strains because (I) strains in a pooled fecal sample can be overgrown by other strains (71) and (II) we did not always isolate all different strains present in a sample. However, since probing the diversity of CSRE/FQRE was also an aim of the study, a trade-off between diversity and preservation had to be made in strain isolation. We nevertheless attempted to isolate all apparently phenotypically different strains per selective plate. If several *E. coli* strains from one fecal sample or even from the same selective plate had the same MP, phenotypic variance had been observed for the strain when growing on Gassner agar or under selective pressure. However, selection for phenotypic variability in an antibiotic environment is not a statistically reliable method for determining the diversity of a population. Döpfer et al. assume that approximately 16 *E. coli* strains must be characterized per fecal sample in order to detect all strains present with a certainty of 95% (72). Another study determined 40 to 80 isolates as necessary to describe the molecular diversity of a reservoir (73). Non-selective sampling of between 0 and up to 10 coliform single colonies per fecal sample was sufficient to identify persisting *E. coli* strains exchanged between free-ranging wild boar and domestic pig populations in Corsica (74). Since the lowest number of CSRE/FQRE isolated per farm was 48, and since an average of 6.7 *E. coli* strains were isolated per pooled fecal sample despite preselection for cephalosporin and fluoroquinolone resistance, it can be assumed that the existing data set represents the diversity of CSRE/FQRE on the sampled farms to a large extent.

This study deliberately did not focus on sampling diseased animals. Many antibiotic resistance surveillance programs, however, target pathogenic species (75). Yet, the analysis of indicator *E. coli* strains allows for a better indication of the overall resistance situation, as apathogenic resistant strains are always present in the microbiome, where they can serve as a resistance reservoir (54) and can lead to reversion of the susceptible phenotype of less frequently present pathogenic strains through horizontal gene transfer. In addition, commensals are subject to some of the same selection pressures as pathogens, including co-selection and cross-resistance. Therefore, our study aimed to describe the existing resistant *E. coli* flora as comprehensively as possible. Moreover, focusing on diseased animals or pathogens would not have been compatible with a longitudinal sampling design and the detection of persistent strains. Finally, the CSRE/FQRE isolated in the present study also encoded virulence factors (data not shown), so it cannot be excluded that they might become more virulent over time and become classified as pathogens in the future.

### Limitations of the study

When interpreting the results, it should be kept in mind that the farms sampled were referred by the Thuringian Pig Health Service, which has an advisory role in supporting farms and veterinary authorities. They mostly attend to farms with herd health or management problems. Despite this pre-selection, the four farms differ fundamentally in their sizes, as well as in the animal health and hygiene management practiced. However, they might depict pigsties in Thuringia with a higher risk of having a problem with antibiotic resistance. Since persistence was only demonstrated for strains preselected on the basis of their cephalosporin and fluoroquinolone resistance, no statement can be made about susceptible persistent strains. To gain insight into these, the antibiotic-free GA bacterial pools would have had to be investigated. Moreover, due to methodological reasons, we cannot make a statement on the quantity of persistent strains because the fraction of persistent strains in the total population of γ-proteobacteria of a pooled fecal sample was not determined. Since strains could also spread first and then pick up resistance, so that resistance was not the original criterion for spreading (76, 77), it should not be assumed that the selected CSRE/FQRE are representative for persistent *E. coli* strains in general. Testing such a hypothesis will require further analysis. A further limitation of the study is the waiver of sampling the mother sows. According to several studies, the intestinal microbiome of piglets is significantly determined by that of the dam (78, 79). However, this study focused on the persistence of resistant bacteria in the farm environment, during and between fattening runs and not on their origin. For this reason and for reasons of overall sample numbers, mother sows were not sampled. At the same time, no sampling with regard to resistant bacteria was carried out before the animals were housed. Therefore, it cannot be ruled out that persistent strains, as defined by their MPs, were already present in the pens of the respective farm before the animals were born, even though the pens or compartments were cleaned beforehand and also consistently disinfected on the conventional farms. In this case, it must be assumed that cleaning and disinfection cannot completely prevent the persistence of resistant strains, as has been shown for poultry farming (44, 80).

Two years after legislation restricting the usage of fluoroquinolones as well as 3rd and 4th generation cephalosporins in animal husbandry was introduced in Germany, resistance to these antibiotic classes is still present in German pigsties. The study shows that CSRE and FQRE vary strongly in the different age groups of the pig production cycles, but that certain strains persist in the farm environment over extended periods of time. Because of their farm specificity, these resistant strains appear to be well-adapted to the particular farm, so their elimination would require either procedures acting in a very general manner against *E. coli* or Enterobacterales or targeted action against the respective specific strains.

## MATERIALS AND METHODS

### Farm selection and general nomenclature

Two organic and two conventional pigsties, located in the Federal State of Thuringia, Germany, were selected based on two criteria inspected in pre-screening tests: (i) Farrow-to-finish pig farms and (ii) the presence of bacteria resistant to 3rd and 4th generation cephalosporins and/or fluoroquinolones in pig feces. The conventional farms practiced all-in-all-out housing, enabling sampling of consecutive fattening runs housed in the same barns. Organic farms, on the other hand, were smaller and kept animals from different fattening runs in different compartments of the same barn during overlapping time periods. We therefore defined consecutive fattening runs on conventional farms as groups of pigs living in the same barn at different times, while on organic farms they consisted of piglets bred at consecutive farrowing times, resulting in a 3-week time lag, but which were kept in parallel in the same barn. During the farm visits, details on farm architecture, organization of animal groups, numbers of animals kept, and general hygiene management were collected for each farm.

### Animal sampling

On farms O1, O2, and C2, pooled fecal droppings were taken from two to four consecutive fattening runs per farm. The following five age groups were sampled: suckling piglets (3.5–6 weeks), weaners (8–12 weeks), early fattening (10–13 weeks), mid-fattening (4.5–5.5 months), and late fattening (> 6.5 months). On organic farms, animals from different fattening runs were occasionally grouped together after a certain age, resulting in merged fattening runs. On farm C1, only suckling piglets were sampled, albeit at a higher frequency.

Fresh fecal droppings from at least five different locations in the pen of the animal group sampled were collected with spatulas (Vivomed GmbH, Solingen, Germany) from the floor and transferred into sterile containers (Neolab Migge, Berlin, Germany). When collecting pooled fecal samples of suckling piglets, efforts were made not to add sow feces to the sample. All samples were kept on ice during transportation to the laboratory and processed within 24 h. On farm C1, sampling was carried out by farm personnel. Samples were mailed to the laboratory and processed within 48 h after sampling. Sampling was carried out from September 2019 until October 2020. Farmers received a cover letter explaining the purpose of the investigation and signed a declaration of consent according to Directive 95/46/EC (General Data Protection Regulation) for taking samples as well as analyzing them for scientific reasons and to ensure confidentiality.

### Sample processing

Counts of bacterial colony-forming units of ceftiofur-, cefquinome-, or enrofloxacin-resistant coliforms were obtained as follows: 1 g of pooled fecal material was resuspended in 9 mL of 0.9% sterile saline, which was further 10-fold diluted, resulting in serial 10-fold dilutions. Aliquots (100 μL) of several successive dilutions per sample were spread by plating onto different solid media in Petri dishes (Gassner agar, designated GA throughout the study; sifin diagnostics, Berlin, Germany) lacking antibiotics or supplemented with either 4 μg/mL ceftiofur (designated GF; Sigma-Aldrich, Zwijndrecht, The Netherlands), 8 μg/mL cefquinome (designated GQ; Carbosynth Holdings Ltd., Berkshire, UK), or 4 μg/mL enrofloxacin (designated GE; Sigma-Aldrich, Zwijndrecht, The Netherlands) and incubated for 18–24 h at 37°C. Hereby, enrofloxacin served as the representative fluoroquinolone, while ceftiofur and cefquinome were chosen to represent 3rd and 4th generation cephalosporins, respectively. The concentrations selected for the GE, GF, and GQ plates were based on clinical breakpoints for various animal species and indications. There were no specific cutoff values because the quantitative analysis considered several different bacterial species, which can grow on Gassner agar, grouping them under the general term “γ-proteobacteria.” Nevertheless, we expect the cephalosporin-selective plates to be sufficiently stringent because *bla*_CTX-M-1_- and *bla*_CTX-M-15_-positive *E. coli* strains display ceftiofur and cefquinome minimal inhibitory concentration values ≥ 32 µg/mL (81). Furthermore, since most quinolone resistance determining region mutations lead to fluoroquinolone minimal inhibitory concentrations significantly above the clinical threshold (82), it can be assumed that growth of fluoroquinolone-resistant *E. coli* will not be inhibited by the GE plates’ enrofloxacin concentration, which was above the clinical breakpoint for resistance. Furthermore, this fluoroquinolone concentration had already been used in another study focusing on FQRE (83). To detect even very low numbers of resistant bacteria, 500 µL of the 10^−1^ dilution was also plated. Plates containing approximately 30–300 colonies were counted, and CFU/g feces calculated. This approach had a minimum detection limit of 20 CFU/g feces. The CFU counts represent the total bacterial growth on the plate, preferably consisting of γ-proteobacteria, i.e., lactose-positive as well as -negative colonies.

### Strain selection

While all pooled fecal samples with colony growth from farm O1 were chosen for isolate retrieval, only certain selective plates were processed from farm C1 due to the large number of samples from this farm. No GQ plates were processed because the CFU percentages on the GF and GQ plates were very similar, suggesting similar or even identical strains. Furthermore, only GE and GF plates from samples with high CFU counts were processed. Following these criteria, the GE (*n* = 16) and/or GF plates (n = 14) from 16 fecal samples were selected for qualitative analysis. From farm C2, only the pooled fecal samples from fattening runs 1 and 2 were processed. At farm O2, an additional suckling piglet sample was analyzed qualitatively. These animals had been added to the animals of fattening run 4, which was not sampled all the way through and not included in the quantitative analysis. Therefore, *E. coli* strains isolated from this sample were only analyzed as part of the suckling piglet age group from farm O2.

Bacterial strains were isolated by picking a single colony of each different coliform phenotype, defined as appearing blue or green on a Gassner agar plate and, thus, representing a presumptive *E. coli*, from each GF, GQ, and GE plate of each sample and freshly streaking them out on a selective plate supplemented with the same antibiotic as the original plate to obtain pure colonies. Colony identity was subsequently confirmed as *E. coli* via matrix-assisted laser desorption ionization-time of flight analysis (threshold for species identification ≥2; [84]).

### *E. coli* genotyping

A modified multiple-locus variable number tandem-repeat (VNTR) analysis (MLVA) protocol (85) was used, which combines the advantages of standard PCR, being easy to perform, rapid, and inexpensive, with a high discriminatory power, superior to that of multilocus sequence typing (MLST) and repetitive element palindromic PCR (REP-PCR) but slightly lower than that of pulsed-field gel electrophoresis (PFGE) (85), as a reproducible typing tool to screen for the presence of chromosomally related or unrelated isolates among the resistant strains. Up to eight *E. coli* colonies were picked from the pure subculture plate, resuspended in 350 µL phosphate-buffered saline (PBS; Life Technologies, Eggenstein, Germany), incubated for 15 min at 99°C, and briefly centrifuged. These cleared lysates were then used for genotyping via MLVA-PCR (85) with the following modifications: Two VNTR loci (ms21 and CNV014) were detected in a duplex-PCR, the other five VNTR loci (RDB1, ms11, CNV001, CNV004 and O157-33) in a separate multiplex-PCR. The *E. coli* strain MG1655 (DSM 18039; GenBank accession no. U00096.3 [86]) was used as a positive control. The duplex PCR was performed in a total volume of 25 µL containing Qiagen Mastermix buffer with HotStarTaq DNA polymerase (Qiagen, Hilden, Germany), 100 nM of each primer (all primers obtained from Eurofins Genomics, Ebersberg, Germany) of the primer pair ECMLV3 (ms21), 240 nM of each primer of the primer pair ECMLV6 (CNV014), and 2 µL of a 1:10 dilution of the cleared lysate. The reaction conditions were 15 min/95°C, followed by 30 cycles of 30 s/94°C, 90 s/55°C, 90 s/72°C, and a final extension step of 10 min/72°C. The multiplex PCR was performed in a total volume of 25 µL containing One *Taq* buffer, GC enhance buffer, 200 µM dNTPs (all New England Biolabs GmbH, Frankfurt/Main, Germany), 50 nM of each primer of the primer pairs ECMLV2 (ms11), ECMLV4 (CNV001), and ECMLV5 (CNV004), 100 nM of each primer of the primer pairs ECMLV1 (RDB1) and ECMLV7 (O157-33), 500 nM of the primer ECMLV1-R2, 0.625 units One *Taq* DNA polymerase (New England Biolabs GmbH, Frankfurt/Main, Germany) and 2 µL cleared lysate. The reaction conditions were 15 min/94°C, followed by 30 cycles of 30 s/94°C, 90 s/58°C, 90 s/68°C, and a final extension step of 10 min/68°C. The resulting PCR fragments were analyzed with 2.5% agarose gels, stained with DNA stain G (Serva Electrophoresis GmbH, Heidelberg, Germany) and their lengths assigned with a 100 bp DNA ladder (New England Biolabs GmbH, Frankfurt/Main, Germany).

### Bioinformatic and statistical analysis

Images of the agarose gels from the MLVA-PCR were analyzed bioinformatically (BioNumerics, version 7.6.3, Applied Maths NV, Sint-Martens-Latem, Belgium). For this purpose, two different experimental categories of the type “fingerprint” were created and interpreted together as a composite data set. The gel images of a MLVA-duplex and a MLVA-multiplex PCR obtained for the same isolate were combined into a single MLVA profile (MP) under equal weighting. The profiles were based on the Dice coefficient of the banding patterns. They were compared using the unweighted pair group method with arithmetic mean as well as a band tolerance of 1.5%, an optimization of 2%, and a tolerance change of 0.5%. To compensate for variations between different gel runs, two strains were assumed to have the same MP if there was at least 98% similarity in their banding patterns. In this case, the strains were assigned to a MLVA cluster. To compare strain diversity across farms, a uniformity index was determined (average number of strains with the same MP), which is higher, the fewer MPs the strains included display. Boxplots were generated in the R statistical environment (v4.2) using ggplot2 (v3.3.6). Phylogenetic trees were displayed using the internet-based program iTOL (87).

## Data Availability

The supporting information can be downloaded at Zenodo (www.zenodo.com) with the following doi: 10.5281/zenodo.14710591.

## References

[1] Holmes AH, Moore LSP, Sundsfjord A, Steinbakk M, Regmi S, Karkey A, Guerin PJ, Piddock LJV. 2016. Understanding the mechanisms and drivers of antimicrobial resistance. Lancet 387:176–187. doi:10.1016/S0140-6736(15)00473-0 26603922

[2] Davies J, Davies D. 2010. Origins and evolution of antibiotic resistance. Microbiol Mol Biol Rev 74:417–433. doi:10.1128/MMBR.00016-10 20805405 PMC2937522

[3] Tran-Dien A, Le Hello S, Bouchier C, Weill FX. 2018. Early transmissible ampicillin resistance in zoonotic Salmonella enterica serotype Typhimurium in the late 1950s: a retrospective, whole-genome sequencing study. Lancet Infect Dis 18:207–214. doi:10.1016/S1473-3099(17)30705-3 29198740

[4] World Health Organization. 2020. 10 global health issues to track in 2021. Available from: https://www.who.int/news-room/spotlight/10-global-health-issues-to-track-in-2021. Retrieved 20 Oct 2023.

[5] Herdman MT, Karo B, Dave J, Katwa P, Freedman J, Do Nascimento V, Kirkbride H, Chattaway MA, Godbole G, Balasegaram S. 2021. Increasingly limited options for the treatment of enteric fever in travellers returning to England, 2014-2019: a cross-sectional analytical study. J Med Microbiol 70:001359. doi:10.1099/jmm.0.001359 34351258 PMC8513630

[6] Cole MJ, Day M, Jacobsson S, Amato-Gauci AJ, Spiteri G, Unemo M. 2022. The European response to control and manage multi- and extensively drug-resistant Neisseria gonorrhoeae. Euro Surveill 27:2100611. doi:10.2807/1560-7917.ES.2022.27.18.2100611 35514307 PMC9074391

[7] Wozniak TM, Dyda A, Merlo G, Hall L. 2022. Disease burden, associated mortality and economic impact of antimicrobial resistant infections in Australia. Lancet Reg Health West Pac 27:100521. doi:10.1016/j.lanwpc.2022.100521 35832237 PMC9271974

[8] Poudel AN, Zhu S, Cooper N, Little P, Tarrant C, Hickman M, Yao G. 2023. The economic burden of antibiotic resistance: a systematic review and meta-analysis. PLoS One 18:e0285170. doi:10.1371/journal.pone.0285170 37155660 PMC10166566

[9] Antimicrobial Resistance Collaborators. 2022. Global burden of bacterial antimicrobial resistance in 2019: a systematic analysis. Lancet 399:629–655. doi:10.1016/S0140-6736(21)02724-0 35065702 PMC8841637

[10] World Health Organization. 2016. Global action plan on antimicrobial resistance. World Health Organization, Geneva, Switzerland.

[11] World Health Organization. 2020. Global antimicrobial resistance surveillance system (GLASS) report: early implementation 2020. World Health Organization, Geneva, Switzerland.

[12] Birkegård AC, Halasa T, Græsbøll K, Clasen J, Folkesson A, Toft N. 2017. Association between selected antimicrobial resistance genes and antimicrobial exposure in Danish pig farms. Sci Rep 7:9683. doi:10.1038/s41598-017-10092-9 28852034 PMC5575052

[13] Van Boeckel TP, Glennon EE, Chen D, Gilbert M, Robinson TP, Grenfell BT, Levin SA, Bonhoeffer S, Laxminarayan R. 2017. Reducing antimicrobial use in food animals. Science 357:1350–1352. doi:10.1126/science.aao1495 28963240 PMC6510296

[14] Lim JM, Singh SR, Duong MC, Legido-Quigley H, Hsu LY, Tam CC. 2020. Impact of national interventions to promote responsible antibiotic use: a systematic review. J Antimicrob Chemother 75:14–29. doi:10.1093/jac/dkz348 31834401 PMC6910191

[15] Roth N, Käsbohrer A, Mayrhofer S, Zitz U, Hofacre C, Domig KJ. 2019. The application of antibiotics in broiler production and the resulting antibiotic resistance in Escherichia coli: a global overview. Poult Sci 98:1791–1804. doi:10.3382/ps/pey539 30544256 PMC6414035

[16] Agency EM. 2022. Sales of veterinary antimicrobial agents in 31 European countries in 2021 – Trends from 2010 to 2021 – Twelfth ESVAC report. Publications Office of the European Union

[17] Ewers C, Bethe A, Semmler T, Guenther S, Wieler LH. 2012. Extended-spectrum β-lactamase-producing and AmpC-producing Escherichia coli from livestock and companion animals, and their putative impact on public health: a global perspective. Clin Microbiol Infect 18:646–655. doi:10.1111/j.1469-0691.2012.03850.x 22519858

[18] Liu YY, Wang Y, Walsh TR, Yi LX, Zhang R, Spencer J, Doi Y, Tian G, Dong B, Huang X, Yu LF, Gu D, Ren H, Chen X, Lv L, He D, Zhou H, Liang Z, Liu JH, Shen J. 2016. Emergence of plasmid-mediated colistin resistance mechanism MCR-1 in animals and human beings in China: a microbiological and molecular biological study. Lancet Infect Dis 16:161–168. doi:10.1016/S1473-3099(15)00424-7 26603172

[19] He T, Wang R, Liu D, Walsh TR, Zhang R, Lv Y, Ke Y, Ji Q, Wei R, Liu Z, et al.. 2019. Emergence of plasmid-mediated high-level tigecycline resistance genes in animals and humans. Nat Microbiol 4:1450–1456. doi:10.1038/s41564-019-0445-2 31133751

[20] Sundqvist M, Geli P, Andersson DI, Sjölund-Karlsson M, Runehagen A, Cars H, Abelson-Storby K, Cars O, Kahlmeter G. 2010. Little evidence for reversibility of trimethoprim resistance after a drastic reduction in trimethoprim use. J Antimicrob Chemother 65:350–360. doi:10.1093/jac/dkp387 19900952

[21] Pakpour S, Jabaji S, Chénier MR. 2012. Frequency of antibiotic resistance in a swine facility 2.5 years after a ban on antibiotics. Microb Ecol 63:41–50. doi:10.1007/s00248-011-9954-0 21997543

[22] Verrette L, Fairbrother JM, Boulianne M. 2019. Effect of cessation of ceftiofur and substitution with lincomycin-spectinomycin on extended-spectrum-β-lactamase/AmpC genes and multidrug resistance in Escherichia coli from a Canadian broiler production pyramid. Appl Environ Microbiol 85:e00037-19. doi:10.1128/AEM.00037-19 31028030 PMC6581166

[23] Abraham S, Kirkwood RN, Laird T, Saputra S, Mitchell T, Singh M, Linn B, Abraham RJ, Pang S, Gordon DM, Trott DJ, O’Dea M. 2018. Dissemination and persistence of extended-spectrum cephalosporin-resistance encoding IncI1-bla_CTXM-1_ plasmid among Escherichia coli in pigs. ISME J 12:2352–2362. doi:10.1038/s41396-018-0200-3 29899511 PMC6155088

[24] Levin BR. 2001. Minimizing potential resistance: a population dynamics view. Clin Infect Dis 33:S161–S199. doi:10.1086/321843 11524714

[25] Ogunlana L, Kaur D, Shaw LP, Jangir P, Walsh T, Uphoff S, MacLean RC. 2023. Regulatory fine-tuning of mcr-1 increases bacterial fitness and stabilises antibiotic resistance in agricultural settings. ISME J 17:2058–2069. doi:10.1038/s41396-023-01509-7 37723338 PMC10579358

[26] Giles M, Cawthraw SA, AbuOun M, Thomas CM, Munera D, Waldor MK, La Ragione RM, Ritchie JM. 2018. Host-specific differences in the contribution of an ESBL IncI1 plasmid to intestinal colonization by Escherichia coli O104:H4. J Antimicrob Chemother 73:1579–1585. doi:10.1093/jac/dky037 29506073 PMC6658847

[27] Wang LYR, Jokinen CC, Laing CR, Johnson RP, Ziebell K, Gannon VPJ. 2020. Assessing the genomic relatedness and evolutionary rates of persistent verotoxigenic Escherichia coli serotypes within a closed beef herd in Canada. Microb Genom 6:e000376. doi:10.1099/mgen.0.000376 32496181 PMC7371104

[28] Cottell JL, Webber MA, Piddock LJV. 2012. Persistence of transferable extended-spectrum-β-lactamase resistance in the absence of antibiotic pressure. Antimicrob Agents Chemother 56:4703–4706. doi:10.1128/AAC.00848-12 22710119 PMC3421869

[29] Lambrecht E, Van Meervenne E, Boon N, Van de Wiele T, Wattiau P, Herman L, Heyndrickx M, Van Coillie E. 2018. Characterization of cefotaxime- and ciprofloxacin-resistant commensal Escherichia coli originating from Belgian farm animals indicates high antibiotic resistance transfer rates. Microb Drug Resist 24:707–717. doi:10.1089/mdr.2017.0226 29148895

[30] Usui M, Sakemi Y, Uchida I, Tamura Y. 2014. Effects of fluoroquinolone treatment and group housing of pigs on the selection and spread of fluoroquinolone-resistant Campylobacter. Vet Microbiol 170:438–441. doi:10.1016/j.vetmic.2014.01.036 24629774

[31] Fleury MA, Mourand G, Jouy E, Touzain F, Le Devendec L, de Boisseson C, Eono F, Cariolet R, Guérin A, Le Goff O, Blanquet-Diot S, Alric M, Kempf I. 2015. Impact of ceftiofur injection on gut microbiota and Escherichia coli resistance in pigs. Antimicrob Agents Chemother 59:5171–5180. doi:10.1128/AAC.00177-15 26077254 PMC4538500

[32] Riley LW. 2020. Distinguishing pathovars from nonpathovars: Escherichia coli. Microbiol Spectr 8. doi:10.1128/microbiolspec.ame-0014-2020 PMC10773148 33385193

[33] Denamur E, Clermont O, Bonacorsi S, Gordon D. 2021. The population genetics of pathogenic Escherichia coli. Nat Rev Microbiol 19:37–54. doi:10.1038/s41579-020-0416-x 32826992

[34] Poirel L, Madec J-Y, Lupo A, Schink AK, Kieffer N, Nordmann P, Schwarz S. 2018. Antimicrobial resistance in Escherichia coli. Microbiol Spectr 6. doi:10.1128/microbiolspec.ARBA-0026-2017 PMC11633601 30003866

[35] Aarestrup FM. 2004. Monitoring of antimicrobial resistance among food animals: principles and limitations. J Vet Med B Infect Dis Vet Public Health 51:380–388. doi:10.1111/j.1439-0450.2004.00775.x 15525370

[36] World Health Organization. 2019. Critically important antimicrobials for human medicine, 6th revision. World Health Organization, Geneva, Switzerland.

[37] Agency EM. 2020. Categorisation of antibiotics in the European Union - Answer to the request from the European Commission for updating the scientific advice on the impact on public health and animal health of the use of antibiotics in animals. Amsterdam, The Netherlands European Medicines Ageny

[38] Schmerold I, van Geijlswijk I, Gehring R. 2023. European regulations on the use of antibiotics in veterinary medicine. Eur J Pharm Sci 189:106473. doi:10.1016/j.ejps.2023.106473 37220817

[39] Bergšpica I, Kaprou G, Alexa EA, Prieto M, Alvarez-Ordóñez A. 2020. Extended spectrum β-lactamase (ESBL) producing Escherichia coli in pigs and pork meat in the European Union. Antibiotics (Basel) 9:678. doi:10.3390/antibiotics9100678 33036406 PMC7600538

[40] Poulin-Laprade D, Brouard JS, Gagnon N, Turcotte A, Langlois A, Matte JJ, Carrillo CD, Zaheer R, McAllister T, Topp E, Talbot G. 2021. Resistance determinants and their genetic context in enterobacteria from a longitudinal study of pigs reared under various husbandry conditions. Appl Environ Microbiol 87:e02612-20. doi:10.1128/AEM.02612-20 33514521 PMC8091121

[41] Dijkshoorn L, Ursing BM, Ursing JB. 2000. Strain, clone and species: comments on three basic concepts of bacteriology. J Med Microbiol 49:397–401. doi:10.1099/0022-1317-49-5-397 10798550

[42] European Food Safety Authority, European Centre for Disease Prevention and Control. 2021. The European Union Summary Report on Antimicrobial Resistance in zoonotic and indicator bacteria from humans, animals and food in 2018/2019. EFSA J 19:e06490. doi:10.2903/j.efsa.2021.6490 33868492 PMC8040295

[43] Storey N, Cawthraw S, Turner O, Rambaldi M, Lemma F, Horton R, Randall L, Duggett NA, AbuOun M, Martelli F, Anjum MF. 2022. Use of genomics to explore AMR persistence in an outdoor pig farm with low antimicrobial usage. Microb Genom 8:000782. doi:10.1099/mgen.0.000782 35344479 PMC9176276

[44] Daehre K, Projahn M, Semmler T, Roesler U, Friese A. 2018. Extended-spectrum β-lactamase-/AmpC β-lactamase-producing Enterobacteriaceae in broiler farms: transmission dynamics at farm level. Microb Drug Resist 24:511–518. doi:10.1089/mdr.2017.0150 28981392

[45] Meissner K, Sauter-Louis C, Heiden SE, Schaufler K, Tomaso H, Conraths FJ, Homeier-Bachmann T. 2022. Extended-spectrum β-lactamase-producing Escherichia coli in conventional and organic pig fattening farms. Microorganisms 10:603. doi:10.3390/microorganisms10030603 35336178 PMC8950372

[46] Schulz J, Kemper N, Hartung J, Janusch F, Mohring SAI, Hamscher G. 2019. Analysis of fluoroquinolones in dusts from intensive livestock farming and the co-occurrence of fluoroquinolone-resistant Escherichia coli. Sci Rep 9:5117. doi:10.1038/s41598-019-41528-z 30914675 PMC6435704

[47] Amsler M, Zurfluh K, Hartnack S, Sidler X, Stephan R, Kümmerlen D. 2021. Occurrence of Escherichia coli non-susceptible to quinolones in faecal samples from fluoroquinolone-treated, contact and control pigs of different ages from 24 Swiss pig farms. Porcine Health Manag 7:29. doi:10.1186/s40813-021-00209-y 33810820 PMC8017651

[48] Hansen KH, Damborg P, Andreasen M, Nielsen SS, Guardabassi L. 2013. Carriage and fecal counts of cefotaxime M-producing Escherichia coli in pigs: a longitudinal study. Appl Environ Microbiol 79:794–798. doi:10.1128/AEM.02399-12 23160131 PMC3568556

[49] Andersen VD, Jensen VF, Vigre H, Andreasen M, Agersø Y. 2015. The use of third and fourth generation cephalosporins affects the occurrence of extended-spectrum cephalosporinase-producing Escherichia coli in Danish pig herds. Vet J 204:345–350. doi:10.1016/j.tvjl.2015.03.014 25935558

[50] Dunlop RH, McEwen SA, Meek AH, Clarke RC, Black WD, Friendship RM. 1998. Associations among antimicrobial drug treatments and antimicrobial resistance of fecal Escherichia coli of swine on 34 farrow-to-finish farms in Ontario, Canada. Prev Vet Med 34:283–305. doi:10.1016/s0167-5877(97)00095-0 9618742

[51] Federal Office of Consumer Protection and Food Safety (BVL). 2020. BVL-Report 14.6 - Bericht zur Resistenzmonitoringstudie 2018: Resistenzsituation bei klinisch wichtigen tierpathogenen Bakterien. Bundesamt für Verbraucherschutz und Lebensmittelsicherheit (BVL), Lebensmittelsicherheit BfVu, Berlin

[52] Mathew AG, Arnett DB, Cullen P, Ebner PD. 2003. Characterization of resistance patterns and detection of apramycin resistance genes in Escherichia coli isolated from swine exposed to various environmental conditions. Int J Food Microbiol 89:11–20. doi:10.1016/s0168-1605(03)00124-7 14580969

[53] de Jong A, Stephan B, Silley P. 2012. Fluoroquinolone resistance of Escherichia coli and Salmonella from healthy livestock and poultry in the EU. J Appl Microbiol 112:239–245. doi:10.1111/j.1365-2672.2011.05193.x 22066763

[54] ECDC / EFSA / EMA. 2021. Third joint inter-agency report on integrated analysis of consumption of antimicrobial agents and occurrence of antimicrobial resistance in bacteria from humans and food-producing animals in the EU/EEA, JIACRA III. Parma, Amsterdam ECDC, EFSA, EMA, Stockholm 10.2903/j.efsa.2021.6712 PMC8243991 34221148

[55] World Health Organization. 2017. Integrated surveillance of antimicrobial resistance in foodborne bacteria: application of a one health approach. World Health Organization, Geneva.

[56] Gao L, Hu J, Zhang X, Wei L, Li S, Miao Z, Chai T. 2015. Application of swine manure on agricultural fields contributes to extended-spectrum β-lactamase-producing Escherichia coli spread in Tai’an, China. Front Microbiol 6:313. doi:10.3389/fmicb.2015.00313 25926828 PMC4396445

[57] Hasan B, Sandegren L, Melhus A, Drobni M, Hernandez J, Waldenström J, Alam M, Olsen B. 2012. Antimicrobial drug-resistant Escherichia coli in wild birds and free-range poultry, Bangladesh. Emerg Infect Dis 18:2055–2058. doi:10.3201/eid1812.120513 23171693 PMC3557866

[58] von Salviati C, Laube H, Guerra B, Roesler U, Friese A. 2015. Emission of ESBL/AmpC-producing Escherichia coli from pig fattening farms to surrounding areas. Vet Microbiol 175:77–84. doi:10.1016/j.vetmic.2014.10.010 25465658

[59] Mathys DA, Mathys BA, Mollenkopf DF, Daniels JB, Wittum TE. 2017. Enterobacteriaceae harboring AmpC (bla_CMY_) and ESBL (bla_CTX-M_) in migratory and nonmigratory wild songbird populations on Ohio dairies. Vector Borne Zoonotic Dis 17:254–259. doi:10.1089/vbz.2016.2038 28165890

[60] Dolejska M, Papagiannitsis CC. 2018. Plasmid-mediated resistance is going wild. Plasmid 99:99–111. doi:10.1016/j.plasmid.2018.09.010 30243983

[61] Fukuda A, Usui M, Okamura M, Dong-Liang H, Tamura Y. 2019. Role of flies in the maintenance of antimicrobial resistance in farm environments. Microb Drug Resist 25:127–132. doi:10.1089/mdr.2017.0371 29708845

[62] Sarmah AK, Meyer MT, Boxall ABA. 2006. A global perspective on the use, sales, exposure pathways, occurrence, fate and effects of veterinary antibiotics (VAs) in the environment. Chemosphere 65:725–759. doi:10.1016/j.chemosphere.2006.03.026 16677683

[63] Noll I, Schweickert B, Tenhagen BA, Käsbohrer A. 2018. Antibiotic consumption and antimicrobial resistance in human and veterinary medicine: an overview of established national surveillance systems in Germany. Bundesgesundheitsbl 61:522–532. doi:10.1007/s00103-018-2724-0 29633033

[64] van Schaik W. 2022. Baas Becking meets One Health. Nat Microbiol 7:482–483. doi:10.1038/s41564-022-01100-4 35365789

[65] Land M, Hauser L, Jun S-R, Nookaew I, Leuze MR, Ahn T-H, Karpinets T, Lund O, Kora G, Wassenaar T, Poudel S, Ussery DW. 2015. Insights from 20 years of bacterial genome sequencing. Funct Integr Genomics 15:141–161. doi:10.1007/s10142-015-0433-4 25722247 PMC4361730

[66] Geurtsen J, de Been M, Weerdenburg E, Zomer A, McNally A, Poolman J. 2022. Genomics and pathotypes of the many faces of Escherichia coli. FEMS Microbiol Rev 46:fuac031. doi:10.1093/femsre/fuac031 35749579 PMC9629502

[67] de Been M, Lanza VF, de Toro M, Scharringa J, Dohmen W, Du Y, Hu J, Lei Y, Li N, Tooming-Klunderud A, Heederik DJJ, Fluit AC, Bonten MJM, Willems RJL, de la Cruz F, van Schaik W. 2014. Dissemination of cephalosporin resistance genes between Escherichia coli strains from farm animals and humans by specific plasmid lineages. PLoS Genet 10:e1004776. doi:10.1371/journal.pgen.1004776 25522320 PMC4270446

[68] Cummings KJ, Rodriguez-Rivera LD, Norman KN, Ohta N, Scott HM. 2017. Identification of a plasmid-mediated quinolone resistance gene in Salmonella isolates from Texas dairy farm environmental samples. Zoonoses Public Health 64:305–307. doi:10.1111/zph.12318 27801549

[69] Marcusson LL, Frimodt-Møller N, Hughes D. 2009. Interplay in the selection of fluoroquinolone resistance and bacterial fitness. PLoS Pathog 5:e1000541. doi:10.1371/journal.ppat.1000541 19662169 PMC2714960

[70] Schaufler K, Semmler T, Pickard DJ, de Toro M, de la Cruz F, Wieler LH, Ewers C, Guenther S. 2016. Carriage of extended-spectrum β-lactamase-plasmids does not reduce fitness but enhances virulence in some strains of pandemic E. coli lineages. Front Microbiol 7:336. doi:10.3389/fmicb.2016.00336 27014251 PMC4794485

[71] Cameron-Veas K, Solà-Ginés M, Moreno MA, Fraile L, Migura-Garcia L. 2015. Impact of the use of β-lactam antimicrobials on the emergence of Escherichia coli isolates resistant to cephalosporins under standard pig-rearing conditions. Appl Environ Microbiol 81:1782–1787. doi:10.1128/AEM.03916-14 25548055 PMC4325139

[72] Döpfer D, Buist W, Soyer Y, Munoz MA, Zadoks RN, Geue L, Engel B. 2008. Assessing genetic heterogeneity within bacterial species isolated from gastrointestinal and environmental samples: how many isolates does it take? Appl Environ Microbiol 74:3490–3496. doi:10.1128/AEM.02789-07 18378649 PMC2423023

[73] Dorado-García A, Mevius DJ, Jacobs JJH, Van Geijlswijk IM, Mouton JW, Wagenaar JA, Heederik DJ. 2016. Quantitative assessment of antimicrobial resistance in livestock during the course of a nationwide antimicrobial use reduction in the Netherlands. J Antimicrob Chemother 71:3607–3619. doi:10.1093/jac/dkw308 27585970

[74] Barth SA, Blome S, Cornelis D, Pietschmann J, Laval M, Maestrini O, Geue L, Charrier F, Etter E, Menge C, Beer M, Jori F. 2018. Faecal Escherichia coli as biological indicator of spatial interaction between domestic pigs and wild boar (Sus scrofa) in Corsica. Transbound Emerg Dis 65:746–757. doi:10.1111/tbed.12799 29322645

[75] European Food Safety Authority (EFSA), European Centre for Disease Prevention and Control (ECDC). 2023. The European Union summary report on antimicrobial resistance in zoonotic and indicator bacteria from humans, animals and food in 2020/2021. EFSA J 21:e07867. doi:10.2903/j.efsa.2023.7867 36891283 PMC9987209

[76] Deschamps C, Clermont O, Hipeaux MC, Arlet G, Denamur E, Branger C. 2009. Multiple acquisitions of CTX-M plasmids in the rare D_2_ genotype of Escherichia coli provide evidence for convergent evolution. Microbiology (Reading) 155:1656–1668. doi:10.1099/mic.0.023234-0 19359321

[77] Reid CJ, Cummins ML, Börjesson S, Brouwer MSM, Hasman H, Hammerum AM, Roer L, Hess S, Berendonk T, Nešporová K, Haenni M, Madec JY, Bethe A, Michael GB, Schink AK, Schwarz S, Dolejska M, Djordjevic SP. 2022. A role for ColV plasmids in the evolution of pathogenic Escherichia coli ST58. Nat Commun 13:683. doi:10.1038/s41467-022-28342-4 35115531 PMC8813906

[78] Callens B, Faes C, Maes D, Catry B, Boyen F, Francoys D, de Jong E, Haesebrouck F, Dewulf J. 2015. Presence of antimicrobial resistance and antimicrobial use in sows are risk factors for antimicrobial resistance in their offspring. Microb Drug Resist 21:50–58. doi:10.1089/mdr.2014.0037 25098762

[79] Chen X, Xu J, Ren E, Su Y, Zhu W. 2018. Co-occurrence of early gut colonization in neonatal piglets with microbiota in the maternal and surrounding delivery environments. Anaerobe 49:30–40. doi:10.1016/j.anaerobe.2017.12.002 29223548

[80] Luyckx K, Millet S, Van Weyenberg S, Herman L, Heyndrickx M, Dewulf J, De Reu K. 2016. A 10-day vacancy period after cleaning and disinfection has no effect on the bacterial load in pig nursery units. BMC Vet Res 12:236. doi:10.1186/s12917-016-0850-1 27760542 PMC5069936

[81] Schink AK, Kadlec K, Schwarz S. 2011. Analysis of bla_CTX-M_-carrying plasmids from Escherichia coli isolates collected in the BfT-GermVet study. Appl Environ Microbiol 77:7142–7146. doi:10.1128/AEM.00559-11 21685166 PMC3194854

[82] Huseby DL, Pietsch F, Brandis G, Garoff L, Tegehall A, Hughes D. 2017. Mutation supply and relative fitness shape the genotypes of ciprofloxacin-resistant Escherichia coli. Mol Biol Evol 34:1029–1039. doi:10.1093/molbev/msx052 28087782 PMC5400412

[83] Römer A, Scherz G, Reupke S, Meißner J, Wallmann J, Kietzmann M, Kaspar H. 2017. Effects of intramuscularly administered enrofloxacin on the susceptibility of commensal intestinal Escherichia coli in pigs (sus scrofa domestica). BMC Vet Res 13:378. doi:10.1186/s12917-017-1260-8 29202759 PMC5715528

[84] Ahmed W, Neubauer H, Tomaso H, El Hofy FI, Monecke S, Abd El-Tawab AA, Hotzel H. 2021. Characterization of enterococci- and ESBL-producing Escherichia coli isolated from milk of bovides with mastitis in Egypt. Pathogens 10:97. doi:10.3390/pathogens10020097 33494211 PMC7909756

[85] Caméléna F, Birgy A, Smail Y, Courroux C, Mariani-Kurkdjian P, Le Hello S, Bonacorsi S, Bidet P. 2019. Rapid and simple universal Escherichia coli genotyping method based on multiple-locus variable-number tandem-repeat analysis using single-tube multiplex PCR and standard gel electrophoresis. Appl Environ Microbiol 85:e02812-18. doi:10.1128/AEM.02812-18 30610078 PMC6414366

[86] Blattner FR, Plunkett G 3rd, Bloch CA, Perna NT, Burland V, Riley M, Collado-Vides J, Glasner JD, Rode CK, Mayhew GF, Gregor J, Davis NW, Kirkpatrick HA, Goeden MA, Rose DJ, Mau B, Shao Y. 1997. The complete genome sequence of Escherichia coli K-12. Science 277:1453–1462. doi:10.1126/science.277.5331.1453 9278503

[87] Letunic I, Bork P. 2021. Interactive Tree Of Life (iTOL) v5: an online tool for phylogenetic tree display and annotation. Nucleic Acids Res 49:W293–W296. doi:10.1093/nar/gkab301 33885785 PMC8265157

